# Developing of nano sized fibrous eutectic silicon in hypereutectic Al–Si alloy by laser remelting

**DOI:** 10.1038/s41598-020-69072-1

**Published:** 2020-07-21

**Authors:** Jaafar Abboud, Jyoti Mazumder

**Affiliations:** 1 Department of Automobile, College of Engineering of Al-Musayab, University of Babylon, Hillah, Iraq; 2 Center for Laser Aided Intelligent Manufacturing, Department of Mechanical Engineering, University of Michigan, 2350 Hayward St, Ann Arbor, MI 48109 USA

**Keywords:** Engineering, Materials science, Nanoscience and technology

## Abstract

Laser surface melting followed by rapid solidification is an effective means to produce very fine microstructures with desirable surface properties because of the high rates of cooling associated with it. In the present study, the effect of rapid cooling on the silicon particle size, distribution, and morphology of hypereutectic Al–17wt.%Si and Al–20wt.%Si alloys have been investigated. A continuous-wave CO2 laser of wavelength 10.6 μm and a Trumpf Yb-YAG disk laser of wavelength 1.030 μm were used with a beam diameter of 1 mm and scanning speeds ranging from 5 to 100 mm/s. Rapid solidification increased the solubility of silicon in aluminum to approximately 5wt% and induced non-equilibrium hypoeutectic microstructures comprising large volume fractions of primary α-Al dendrites and ultrafine Al–Si eutectic of lamellar morphology. Both α-Al dendrites and the silicon particle sizes were significantly reduced from micron to nanoscale level. The morphology of silicon particles is modified from massive polygonal and plate-like to a mixture of fine flakes with round corners, feathery and fibrous, or a coral-like and thread-like structure. The eutectic silicon size and the interlamellar spacing were reduced to 30 and 10 nm, respectively. Furthermore, most of the silicon crystals in the eutectic region and the aluminum dendrites contained a significant number of twins which were considered as an essential contributor to the mechanism of growth and branching. Microhardness values increased two to threefold due to the refinement of the microstructural constituent.

## Introduction

Al–Si casting alloys have received considerable interest as candidate materials in the automotive and aerospace applications due to the high specific strength, relatively low coefficient of thermal expansion, good wear resistance, and excellent fluidity^1,2^. Al–Si alloys used for engine block can be divided into three categories based on their silicon percentage: hypoeutectic (< 12. wt%), eutectic (12 wt% to 12.6 wt%), and hypereutectic (> 12.6 wt%)^1^. The microstructure of the hypoeutectic Al–Si cast alloy usually consists of a primary phase (α-Al) and eutectic mixture of Al–Si. The eutectic silicon crystallizes under conventional solidification conditions into a course, plate-like morphology. However, under conventional solidification conditions, the Si phase in cast Al–Si alloys often exhibits a coarse microstructure that leads to poor mechanical properties^3,5^^.^ When Si content is more than 12%, primary Si particles begin to crystallize as a massive phase with angular morphology which leads to a further reduction in ductility and toughness. Therefore, to use eutectic and hypereutectic Al–Si alloys in engineering applications, the morphology, shape, and size distribution of the silicon need to be modified and refined.

Modification of the Al–Si eutectic has been achieved in different ways such as by the addition of certain elements^6–12^, rapid cooling^13–16,32–34^, heat treatment^17^ and by controlling solidification conditions^18^. Several modifiers are known (e.g., strontium, sodium, antimony, barium, erbium, and cerium), of which strontium is the most employed in the Al alloy industry. The addition of a few hundred parts per million of Sr modifies the eutectic Si morphology from a coarse plate-like into a fine fibrous one and has a beneficial effect on both strength and ductility. In hypereutectic compositions, phosphorus is added to the molten alloys. Investigations have shown that retained trace concentrations as low as 0.0015 through 0.03% P are effective in achieving the refined structure. The effects of different concentrations of Ba, Ca, Y, and Yb on the eutectic arrest have been studied^11^ to deduce that all of these elements cause a depression of the eutectic point. However, Ba and Ca resulted in fibrous eutectic while Y and Yb resulted in refined plate-like eutectic silicon.

Laser surface melting (LSM) treatments have been studied extensively to obtain a very fine near-surface microstructure through nonequilibrium solidification and hence improve surface-related properties such as wear, corrosion, and erosion^20–24^. The cooling rate during solidification was estimated within the range of 104 to 107 K/s, much faster than the conventional solidification rates which are 10^2^ °C/s or less; such high cooling rates have a significant effect on the solidified microstructure such as refinement of the microstructure, increased solid solubility and minimized segregation. Under some conditions, an amorphous structure is produced. The practical applications of laser surface melting is numerous in terms of improving resistance to wear, corrosion, erosion, and oxidation^32^.

Recently there has been growing interest in the use of eutectic alloys such as Al–Si and Al–Cu alloys in engineering applications. Most important eutectic alloys are composed of two phases. Such materials can exhibit outstanding mechanical and electrical properties because their microstructures act as natural or in situ composite material. This has led to an extensive theoretical and experimental study of the relationship between microstructure and solidification conditions.

Size, morphology, and distribution of eutectic silicon are vital microstructural parameters determining the mechanical properties of Al–Si alloys. It is well known that the as-cast unmodified eutectic silicon has an undesired plate-like morphology, which is the stress concentrator reducing strength, ductility, and fatigue strength. This investigation aims at studying the effects of cooling rate on the microstructure of hypereutectic Al–Si alloys with a view to refine and modify the silicon eutectic particles and hence improve surface-related properties such as wear and strength.

## Materials and methods

### Materials

Two substrate specimens of hyper-eutectic composition were used in the present work (Table 1). The first substrate Al-17wt.%Si alloy was in the form of a plate of 55 × 30 × 6 mm cut into many equal thickness samples. The other Al–20wt%Si specimen was in the form of a cylinder of dia. 18 mm. and length 180 mm. It was made by a conventional casting process by melting 50 mm dia. aluminium cylinder of purity of 99.7% with high purity silicon (99.9%). The weighed mixture of aluminum and silicon enveloped in aluminium foil was taken in a clay bonded silicon carbide crucible and heated until melting. After ensuring homogeneity by proper mixing, the melt was poured into a grey cast iron mould preheated to 300 °C to get a cylindrical casting 18 mm in diameter and 180 mm in length. Before laser treatment, the samples were cut into many pieces of equal size followed by surface grinding with 600 grade emery paper, cleaned with alcohol and dried.

**Table 1:** Chemical composition of the as–received Al–Si alloys.

Alloy	Shape	Si	Mn	Mg	Fe	Al
Al–17wt.%Si	Plate—6 mm thick	17.0	0.04	0.34	0.1	Bal
Al-20wt.%Si	Cylinder—18 mm diameter	19.5	–	–	0.3	Bal

### Laser melting

Two different types of lasers were used. A continuous-wave CO_2_ laser of wavelength 10.6 μm and a solid-state disk laser (Trumpf Laser HLD 4,002) of wavelength 1.030 um with a beam diameter of approximately 1 mm were used. Before laser treatment, the sample surface was ground with 600 grade SiC paper, cleaned with alcohol, and then fixed on a copper plate below the laser head to increase the cooling rate during solidification. The sample surface was irradiated by the laser beam moving with scan speeds ranging from 5 to 100 mm/s. Argon shielding gas (flow rate 20 l/min) was used during the laser melting process to prevent oxidation. Some Al–Si samples were immersed in liquid nitrogen before laser treatment to increase the cooling rate. Table 2 lists the laser processing parameters used in the present work.

**Table 2:** Laser processing parameters used in the present work.

Sample no.	Track no.	Laser power W	Laser spot diameter mm	Scanning speed, mm/s	Cooling condition
A	1	2,500—Yb-YAG laser	1	5	Ar
A	2	1,800—CO2 laser	1	10	Ar
A	3	1,800—CO2 laser	1	25	Ar
A	4	2,500—Yb-YAG laser	1	100	Ar
B	1	2,500—Yb-YAG laser	1	5	LN
B	2	2,500—Yb-YAG laser	1	50	LN
B	3	2,500—Yb-YAG laser	1	100	LN

*Ar* Argon, *LN* Liquid Nitrogen.

### Sample analysis

After laser melting, transverse sections were cut from all the laser melted samples for dimensional measurements, microstructural analysis, and microhardness measurements. A standard metallographic procedure of grinding, polishing, and etching with Keller solution for 5 s was used in all cases. Optical microscope, scanning electron microscope (TESCAN MIRA3 FEG SEM), transmission electron microscope (TEM, JEOL 2010F AEM operated at 200 kV), and XRD unit with Cu k_α_ radiation were used to characterize the microstructure and compositional analysis. For TEM, samples 3 mm in dia. were mechanically punched out. Thereafter, the samples were thinned by Precision Ion Polishing System (PIPS) model 691 operated at beam energy 3.5 kV to minimize the beam effects on the microstructure.

### Indentation hardness

In addition to the conventional microhardness measurement, the indentation hardness experiment was also conducted at room temperature on a TI 950 Hysteron Triboindenter equipped with a diamond tip of three-sided pyramid Berkovich probe of approximately 50–200 nm radius. A high load transducer was selected to perform the indentation in load control mode, with a peak load of 850 µN.

### X-ray diffraction

X-ray diffraction study for phase analyses and the lattice parameter measurements was performed using MiniFlex 600 model diffractometer operated at 40 kV and 15 mA. The X-ray diffraction patterns were obtained utilizing Cukα radiation with a wavelength of 1.5406 Å. For phase identification, measurements were scanned for a wide range of diffraction angles (2 *thetas) *ranging from 20 to 100 degrees with a scanning rate of 5o/min.

## Results

### General shape of the laser melted zone

**Figure 1:**
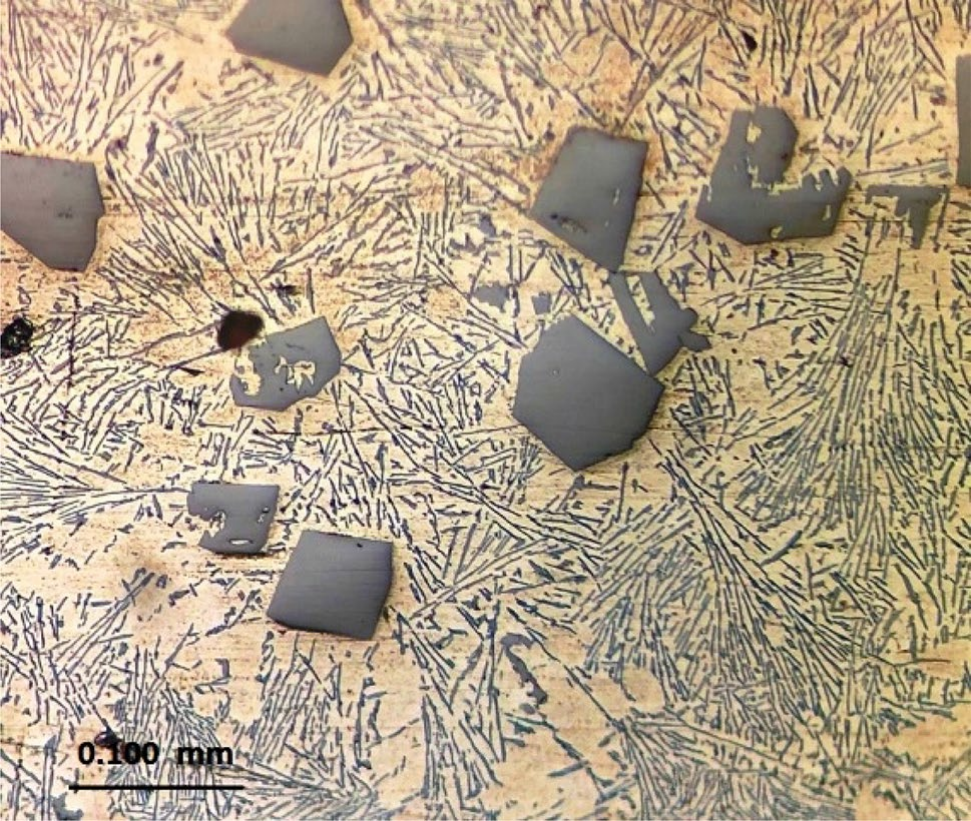
Typical optical microstructure (Optical) of the as-cast Al-20wt.% alloy consisting of massive primary silicon embedded in needles or plate-like Al–Si eutectic.

The microstructure of as-cast Al-17wt.%Si and Al-20wt.%Si alloys were similar and consisted of primary silicon embedded in Al–Si eutectic (Fig. 1). Some aluminum dendrites were also observed at different locations adjacent to the primary silicon. The average size of the primary Si particles ranged between 70 and 100 μm. The eutectic mixture, though, is non-lamellar in form as the classical eutectic form, it appears, in section, to consist of separate flake-like or plate-like Si particles of various lengths and widely spaced; the average widths and spacing are 2 μm and 10 μm, respectively. The microhardness value of the matrix excluding the primary silicon depends on the eutectic spacing and ranged between 57 HV for widely spaced (10 µm) to 70 HV for closely spaced (2 µm).

**Figure 2:**
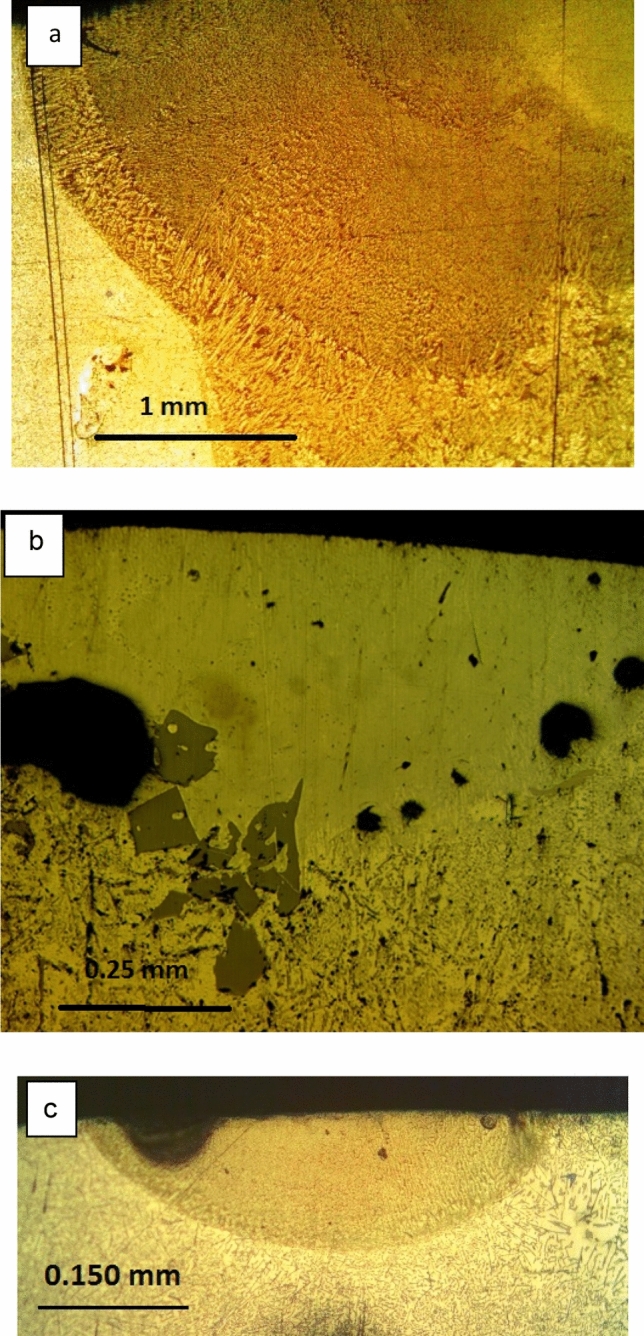
Optical micrographs showing transvers sections of the LMZ of Al-17wt.%Si alloy processed at different laser type, powers and scanning speeds. **a** 2,500 W, 5 mm/s, **b** 1,800 W, 10 mm/s, and **c** 2500 W and 100 mm/s.

Figure 2a–c show transverse sections of three laser melted zone of Al-17wt.%Si alloy processed at different laser types, powers, and scanning speeds. It is clear that with increasing scanning speeds, both the width and the depth were reduced. The minimum melted zone width and depth were 1 mm and 0.16 mm, respectively. Due to the high power and slow scanning speed used the melted zone has a keyhole shape extended to a depth of 3 mm with a big pore adjacent to the root of the keyhole (Fig. 2a) while the laser melted zones processed at high speed produced a conduction limited zone (Fig. 2b, c). Increasing scanning speed leads to reduced heat input and consequently decreases both the melted width and depth.

Figure 2a shows cross-section of the laser melted zone of Al-17wt.%Si alloy processed with TRUMPF laser operated at 2,500 W laser power, 1 mm beam diameter, and 5 mm/s scanning speed. Due to the high-power density used, the melted zone has a keyhole shape extended to a depth of 3 mm with a big pore adjacent to the root of the keyhole. It can be observed that the microstructure is very fine dendritic, and all the primary Si particles and silicon eutectic needles and flakes were completely dissolved and were replaced by fine dendritic structure. The dendrites near the solid/liquid (S/L) interface have columnar structures radially oriented which indicate heat flow normal to the S/L interface due to the high-temperature gradient at the interface. However away from the interface, columnar dendrites tend to change to equiaxed and finer grains. No crack has been observed.

Figure 2b is a cross-section of the same alloy processed with CW CO2 laser operated at 1,800 W laser power, 1 mm beam diameter and 10 mm/s and 100 mm/s scanning speed while Fig. 2c is the structure of the same alloy processed with TRUMPF laser at 2,500 W and 100 mm/s scanning speed. A significant decrease in the width and depth of the melted zone was observed due to the reduced heat input. However, the cooling rate in these samples is expected to be higher due to the small size of the melted volume.

### Microstructure

#### Al-17wt.%Si alloy

**Figure 3:**
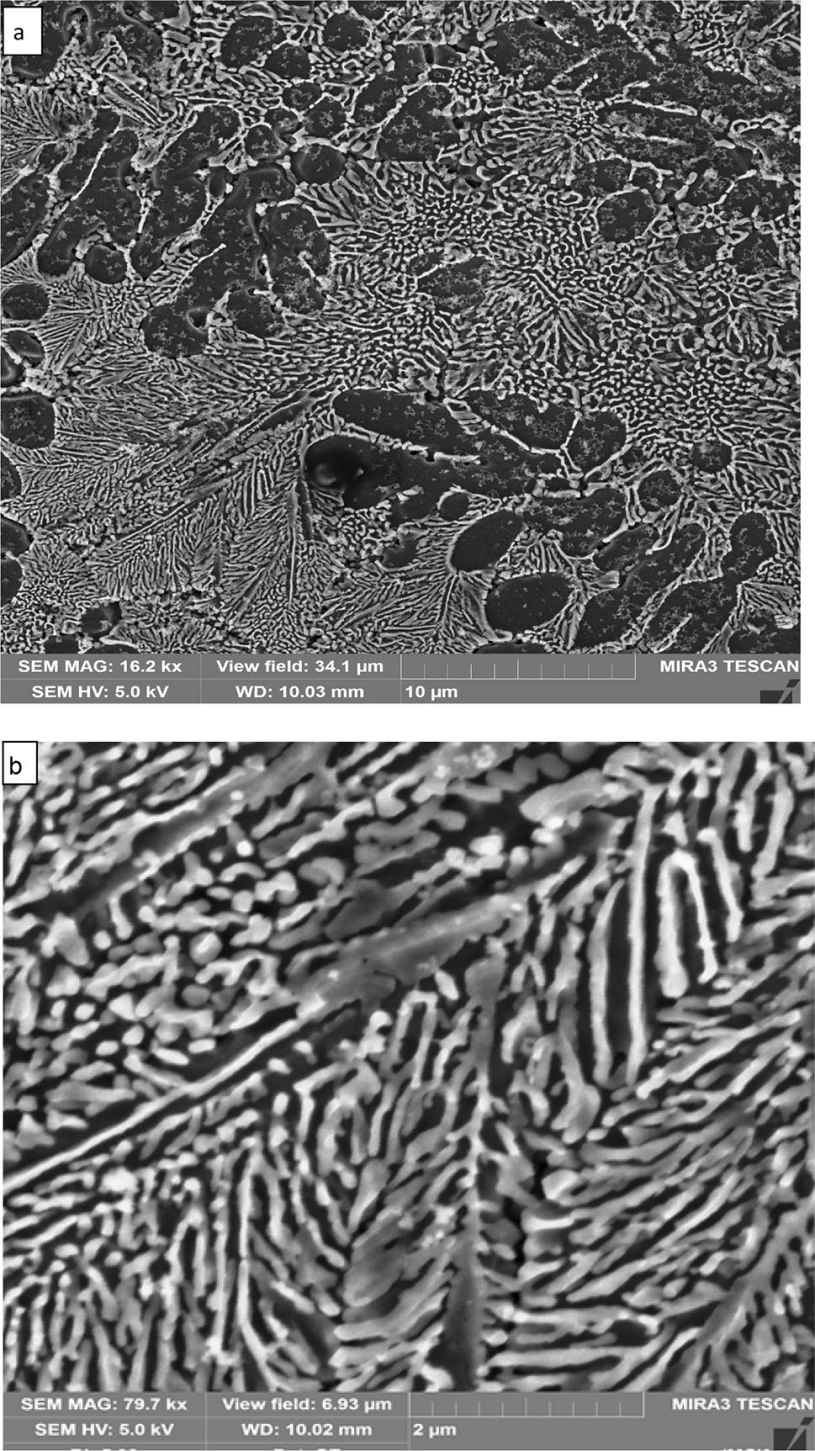
**a** Secondary electron micrographs of Al-17wt.%Si alloy processed at power 2,500 W and scanning speed 5 mm/s, melted depth 3 mm. (black regions are Al dendrites and white areas are silicon. **b** SEM showed a feathery morphology of the Al–Si eutectic region.

More details about the laser melted zone microstructure of the specimen shown in Fig. 2a are presented in Fig. 3a,b. The dendrites (which appear dark) are α-Al phase grown epitaxially from the substrate; within these dendrites, silicon particles of various sizes and shapes can be seen and some of these particles are less than 10 nm in size; these fine silicon particles may be precipitated during cooling. Measurement of the α-Al dendrite sizes/radius from the SEM image showed that it ranged between 2 and 3 µm with secondary dendrite arm spacing (SDAS) less than 2 µm which suggests a high degree of cooling rate achieved during solidification. The interdendritic region showed a lamellar Al–Si eutectic of a very fine scale and various morphologies ranging from a feathery, curved flaky with a rounded corner to straight and elongated rods with a spherical head. The silicon eutectic shape deviated from a faceted morphology toward a non-faceted shape with severe branching (Fig. 3b). The average interlamellar eutectic spacing λ as measured from the SEM image ranged from 100 nm in the lower part decreasing to 50 nm in the central and upper regions.

**Figure 4:**
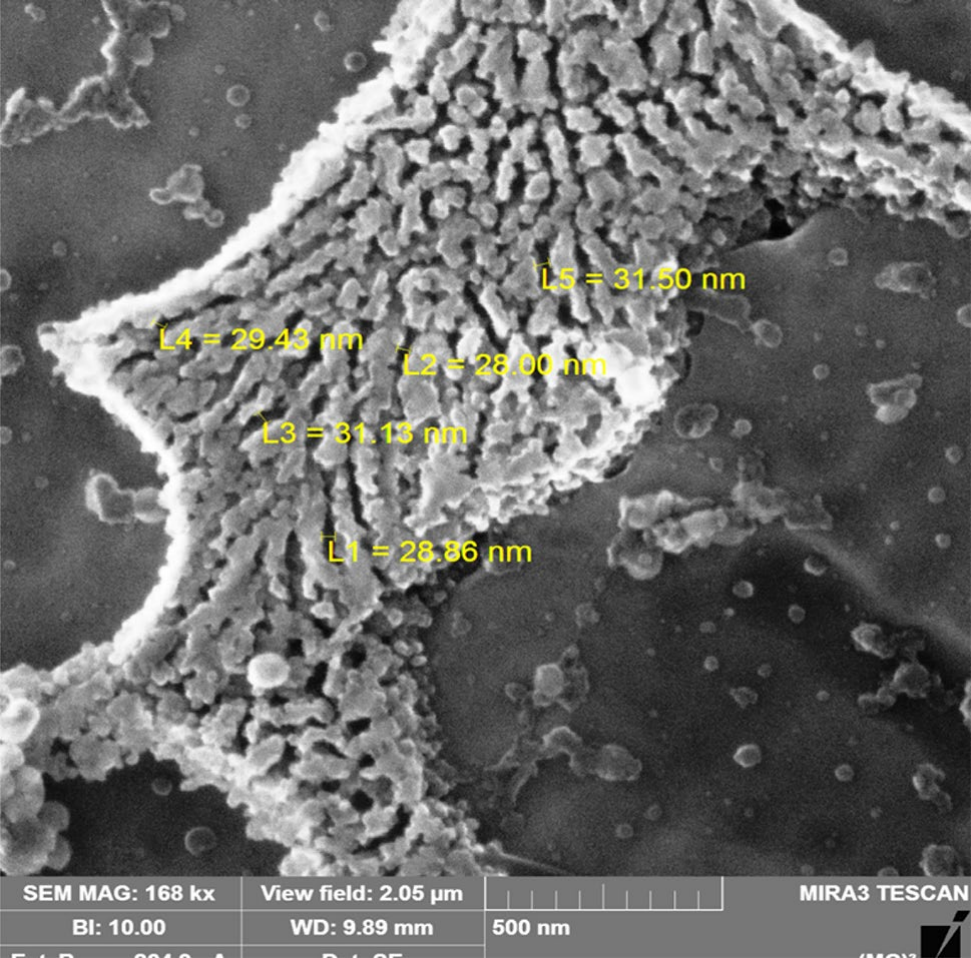
SEM micrograph of the LMZ of Al-17wt.%Si alloy processed with CW CO2 laser (1800 W and 10 mm/s). Average λ = 30 nm.

Figure 4 shows SEM micrograph of the sample processed with CW CO2 laser (1,800 W and 10 mm/s (see Fig. 2b). It shows agglomerated silicon particles of broad flaky shape in the interdendritic region while the dendrites showed many silicon particles of different sizes. The average interlamellar eutectic spacing λ is 30 nm.

**Figure 5:**
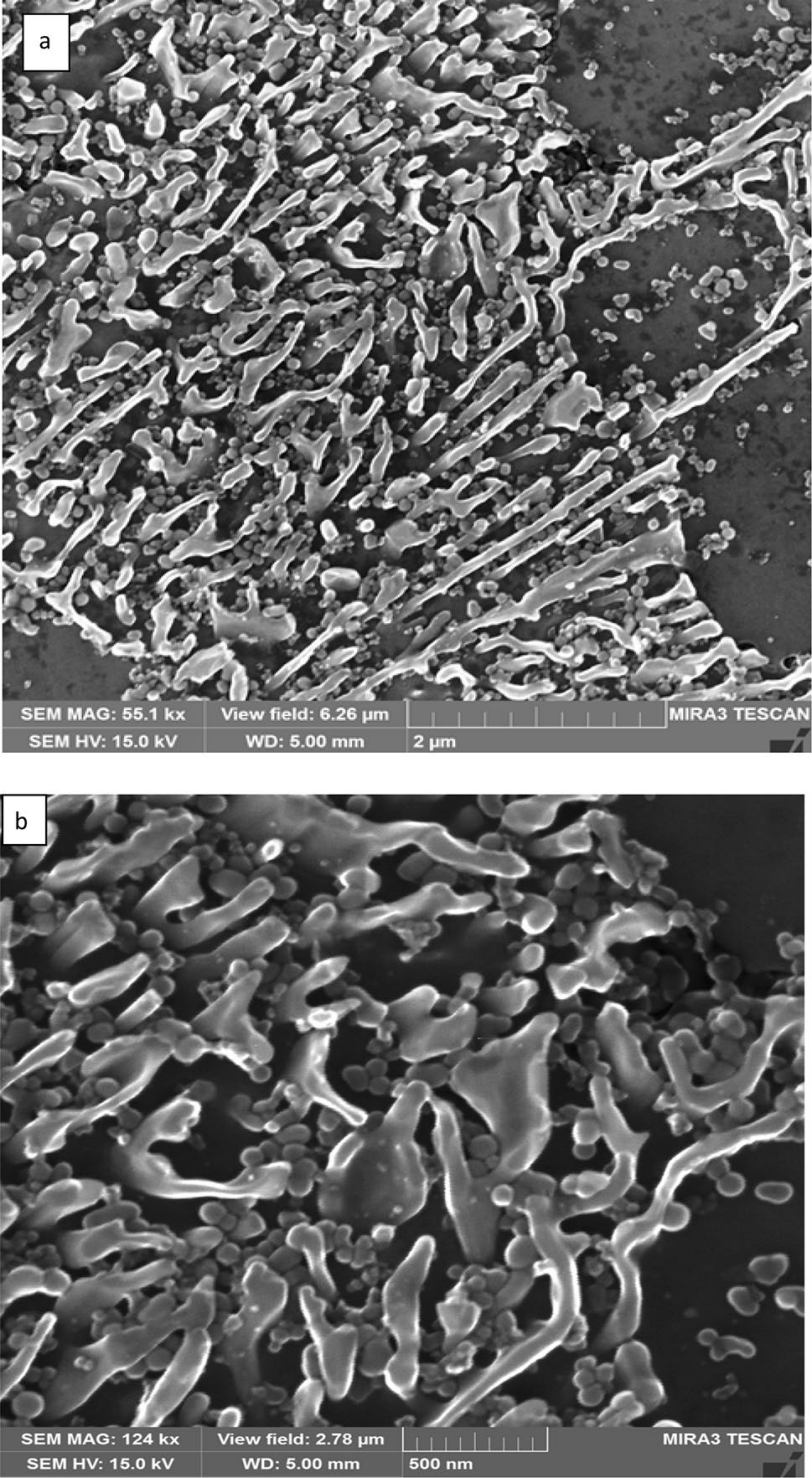
**a**, **b** Secondary electron micrographs of the LMZ processed at the fastest speed 100 mm/s showing flaky fibrous and small rounded and agglomerated silicon crystals.

Figure 5a,b show the microstructure of laser melted zone processed at the fastest scanning speed of 100 mm/s (optical micrograph shown in Fig. 2c). A remarkable refinement of the Al dendrite size and spacing as well as eutectic silicon size, and λ is observed. The Si particles have thin, curved flaky with rounded corners and a tendency to elongate in different directions; also, fine and rounded silicon particles are seen distributed throughout the melted zone (see Fig. 5). It appears that with increasing scanning speed and cooling rate, the silicon particles exhibit greater branching. From the above results, it appears that rapid cooling of Al-17wt.% Si produces a hypoeutectic structure with a high volume fraction of α Al dendrites, reduces the size and SDAS of the dendrites, produces fine silicon particles with fibrous morphology, and possibly increases the solubility of Si in aluminum. However, due to the presence of fine Si particles embedded in Aluminium dendrite, it is very difficult to measure the amount of silicon dissolved in α-Al dendrites.

#### Al-20wt.%Si alloy

**Figure 6:**
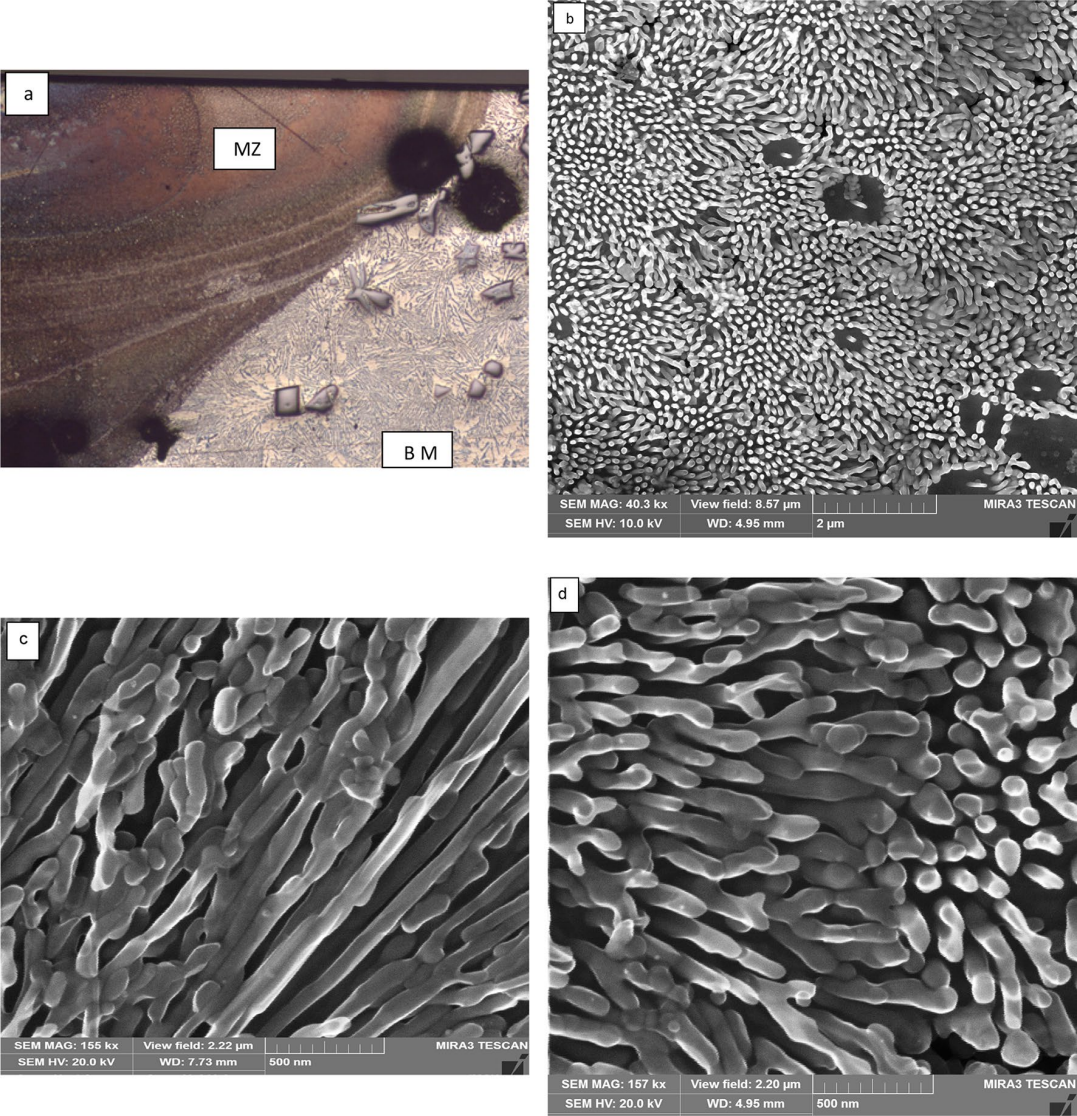
**a** Optical micrograph of transverse section of LMZ of Al-20% Si alloy immersed in liquid nitrogen and laser remelted at 2,500 W and 5 mm/s, **b** SEM micrographs showing α-Al dendrites and lamellar Al–Si eutectic, **c**, **d** SEM micrographs showing the morphology of silicon crystals in the eutectic zone at high magnification.

**Figure 7:**
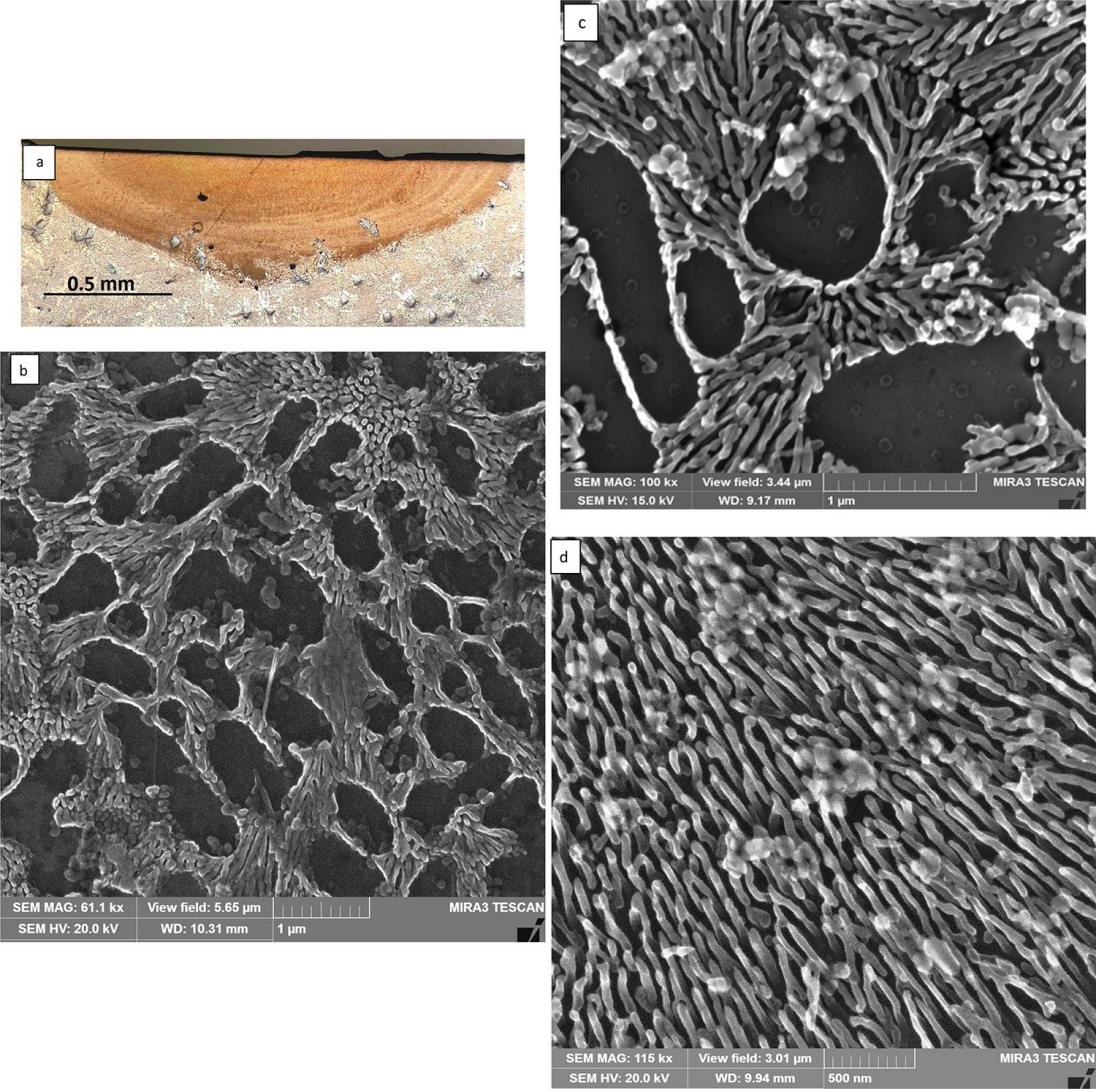
**a** Light optical microstructure of the LMZ of Al-20wt.%Si immersed in liquid nitrogen and laser melted at 50 mm/s scanning speed, **b**, **c** are SEM micrographs taken from the central and upper part of the LMZ (**d**) SEM micrographs taken from the lower part of LMZ showed predominantly thread like fibrous Al–Si eutectic.

To produce more refinement in the Si size and spacing, Al-20wt.%Si sample was selected for laser melting by TRUMPF laser. The samples were immersed in liquid nitrogen before laser melting to get enhanced cooling. Figure 6 exhibits the microstructure of Al-20wt.%Si alloy processed at 2,500 W, and 5 mm/s scanning speed. The melted depth was 3 mm. The SEM micrographs reveal that there is an increase in the volume fraction of the lamellar eutectic and a reduction in the volume fraction of α-Al dendrite. The interlamellar spacing λ was less than 30 nm (Fig. 6b,c). Figure 7 is the microstructure of LMZ of Al-20% Si immersed in liquid nitrogen and laser melted at 50 mm/s. The corresponding melted depth is 0.45 mm. The microstructure showed silicon particles of fibrous shape, highly directional, and closer to regular lamellar eutectic with a uniform λ spacing (20 nm) and less tendency to side branching. EDS analysis performed on the bulk sample of the melted zone of Al-20wt.%Si alloy showed an average silicon content of 19.5 wt% which is very close to the nominal composition of the alloy.

**Figure 8:**
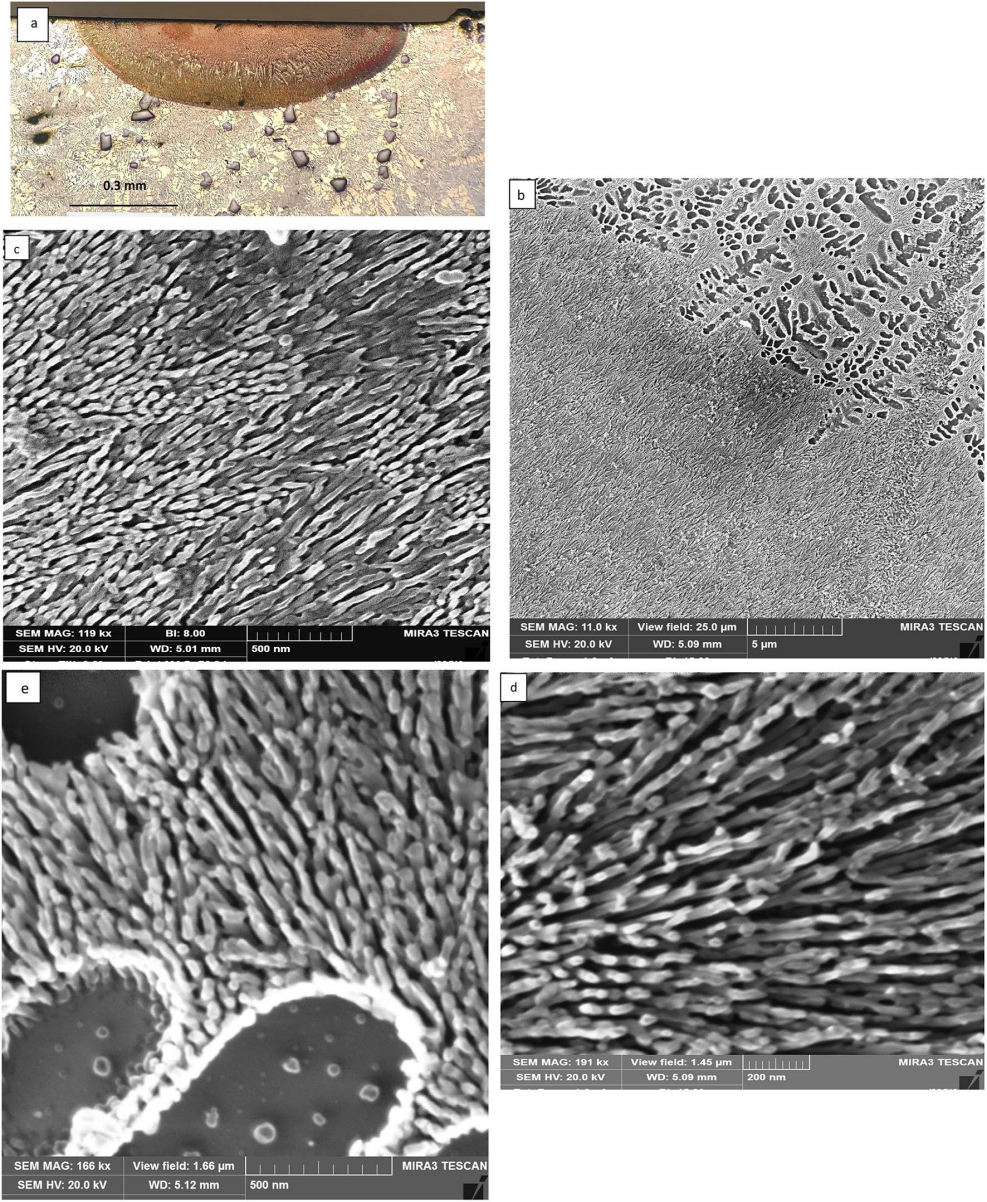
**a** Optical micrograph of laser melted zone of Al-20wt.%Si alloy immersed in liquid nitrogen followed by laser melting at fast speed 100 mm/s, **b **SEM micrograph showing a magnified view of the area in (**a**). SEM micrographs showing (**c**, **d**) thread-like fibrous silicon crystals in the lower part of the LMZ and **e** fine α-Al dendrites and eutectic in the upper part of the LMZ.

Figure 8a shows the optical micrograph of the LMZ processed at the fastest speed of 100 mm/s. It revealed that columnar grains have a directional growth normal to the boundary interface. It seems that heat loss through the substrate has led to more rapid cooling via the substrate than through convection and radiation. This has led to the directional growth of the grains to the cooling direction and subsequently to the formation of columnar grains. SEM examination near the melted zone interface and above showed a predominantly elongated colony of eutectic with a direction normal to the interface and has a thread-like shape (Fig. 8b–d). Figure 8c,d which was taken from the lower part of the melted zone exhibited a thread-like eutectic structure with a significant reduction of the interlamellar spacing λ to less than 10 nm. Figure 8e showed the central part of the melted zone which displays some α–Al dendrites of 500 nm spacing surrounded by eutectic. In summary, silicon crystals in the laser melted zone at various processing conditions showed very fine fibrous plates morphology with irregular changes in width and thickness over the length direction. More details about the internal structure of the silicon particles in the eutectic and within the α- dendrites is presented below:

#### TEM examination

**Figure 9:**
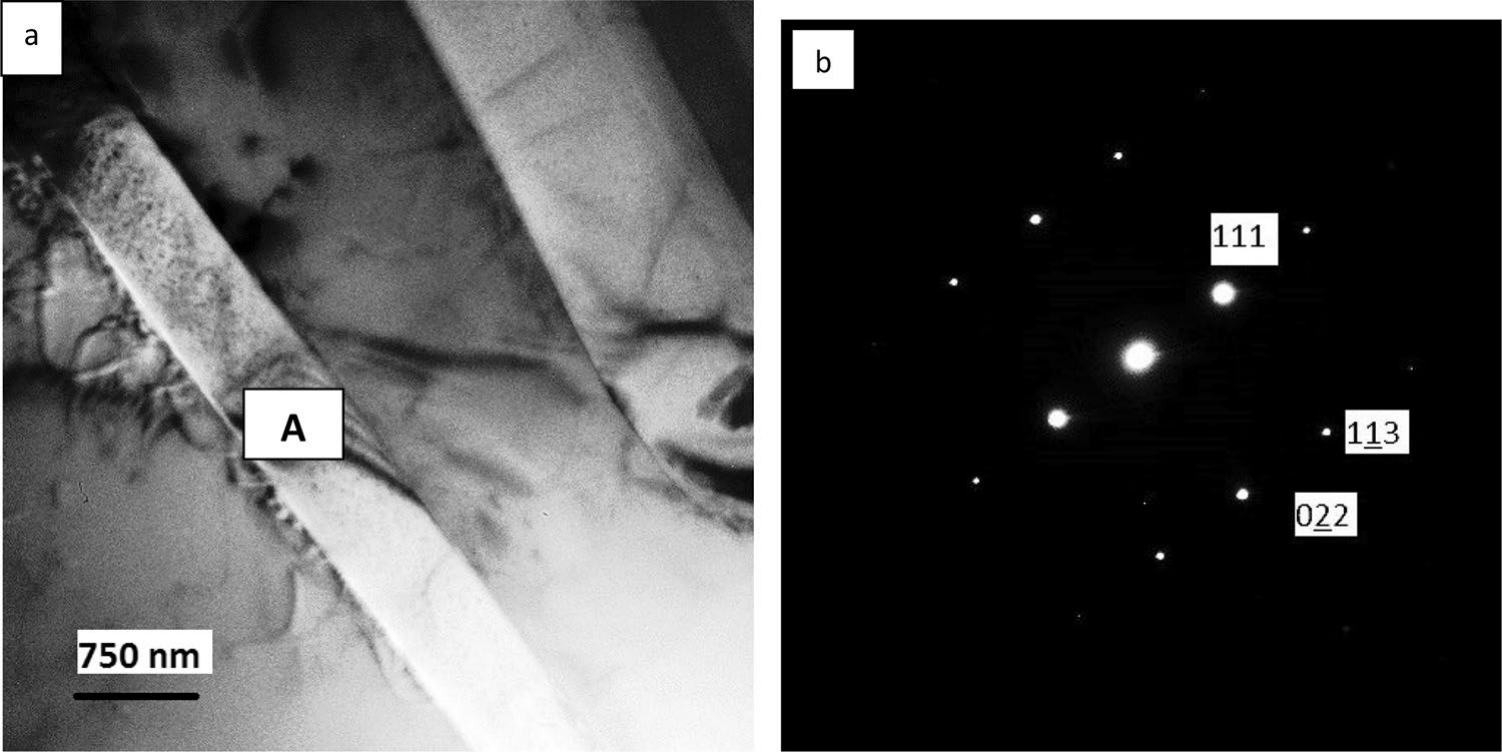
**a** Bright field TEM micrograph showed eutectic Si plate and Al matrix in the as- received Al-17wt.%Si alloy, **b** corresponding diffraction pattern of the Si particle (region A), zone axis [211].

**Figure 10:**
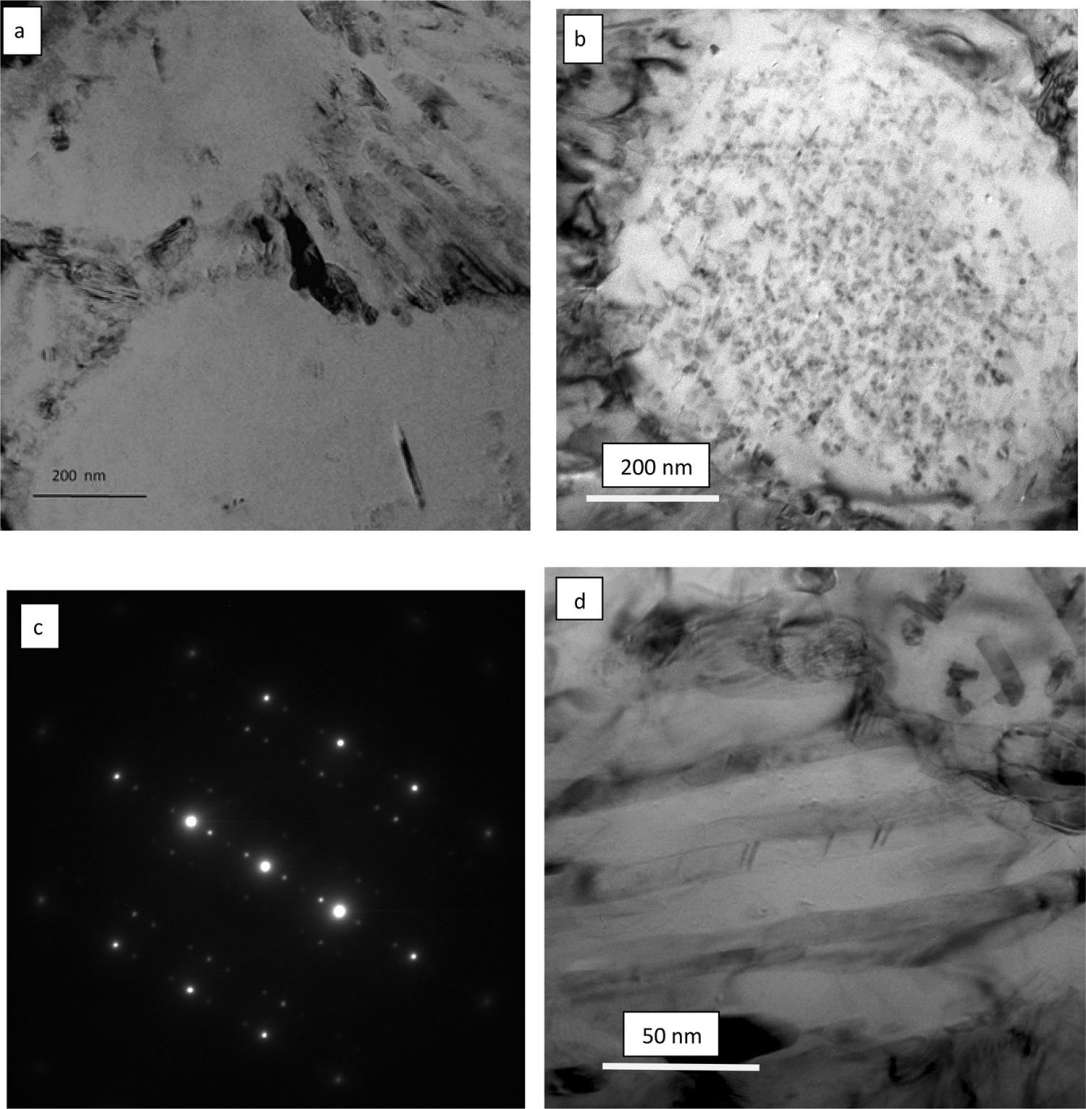
TEM micrographs from the LMZ of Al-17wt.%Si alloy showing **a** the α-Al dendrite and Al–Si eutectic, **b** the α-Al dendrite contained very fine silicon crystals, extra spots were originated from the very fine and twinned silicon particles **c** SADP from α-Al dendrite in (**b**); **d** TEM micrograph showing silicon morphology in the eutectic region.

**Figure 11:**
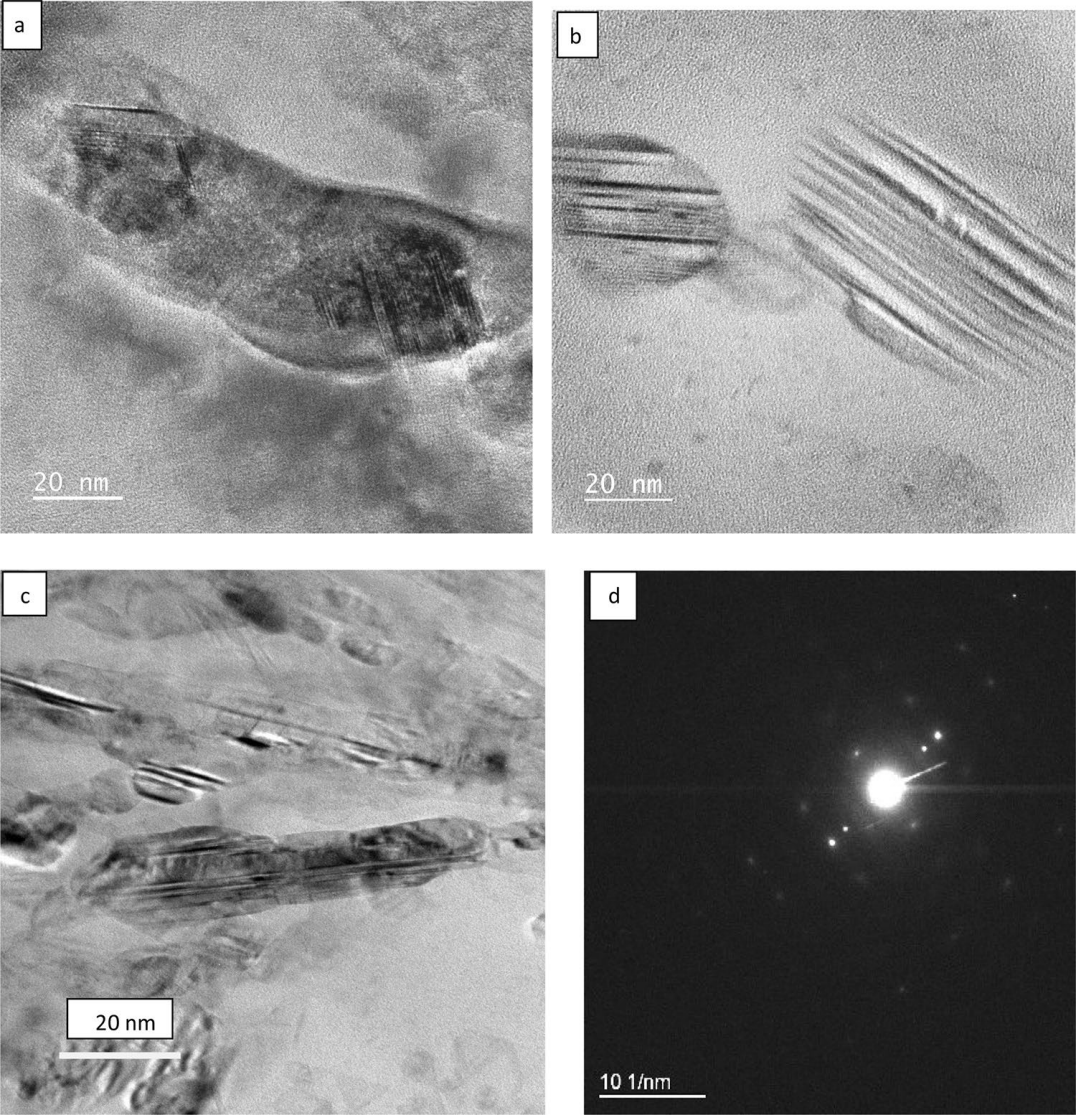
**a**–**c** bright field TEM images showing a region containing an exceptionally high twin density in the silicon particle of the LMZ. **d** Diffraction pattern from the long silicon particle in (**c**), zone axis [110].

TEM examination of the as-received and laser melted zone of the Al-17%Si alloy is presented in Figs. 9, 10, and 11. Figure 9a shows a twins-free silicon plate with the corresponding diffraction pattern from the center of the plate. Figure 10a shows the α-Al dendrite and the lamellar eutectic structure. The dendrites contain a huge amount of silicon particles of various sizes ranging from 30 nm to as small as 5 nm (Fig. 10b). Figure 10c is the corresponding diffraction pattern from the aluminum dendrite and the small Si crystals; the strong diffraction spots belong to Al while the extra spots belong to the Si. Figure 10d clearly shows the irregular shape of the Si eutectic. More details about the internal structure of the Si crystal are shown in Fig. 11. The aluminum dendrites contain a high density of dislocations while the Si crystals have many twins (Figs. 10b, 11a–c). The spacing of twins ranged between 1 and 4 nm (Fig. 11a,b); some of these twins are partially covering the whole width of the silicon particle (Fig. 11c).

**Figure 12:**
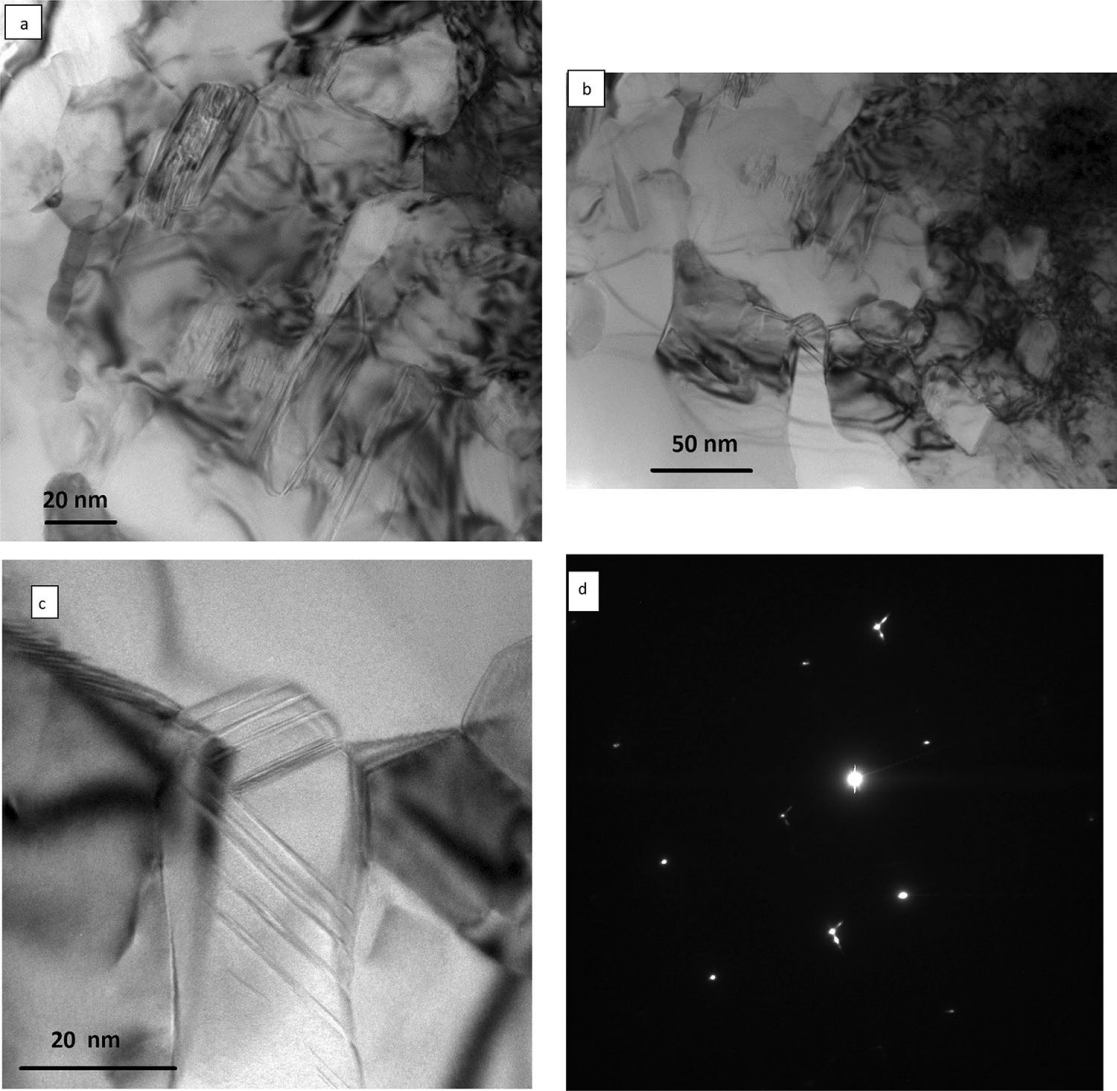
**a**–**c** Bright field TEM micrographs showing the structure of the silicon in laser melted zone of Al-20wt%Si alloy, 2500 W, 1mm, and 100 mm/s. **d** corresponding diffraction pattern from the twined silicon particle in (**c**), zone axis $\left[ {2\underline{{11}} } \right]$.

EDS analysis was performed at different locations in the aluminum dendrites excluding the Si particles showing a maximum value of 5wt%Si. This value is too high and is 10 times higher than the normal solubility of silicon in aluminum under low cooling conditions. Such a high value of dissolved Si indicates that a high cooling rate has increased the solubility of silicon in aluminum.

A similar feature and structure of the eutectic silicon (Fig. 12a–d) was observed in the TEM in the laser melted zone of the Al-20% Si alloy. A high density of twins and dislocations were also seen. Some of these twins extended in the longitudinal and others in the transverse directions. The corresponding diffraction pattern from these twined Si areas showed streaks and tilting was required to get the exact zone axis.

#### Cooling rate

The result of the microstructural analysis above leads to the observation that as the cooling rate increased, the secondary dendrite arm spacing (SDAS) decreased. This is because with the increased cooling rate, both the critical nucleation radius and the nucleation activation energy decrease, and the nucleation rate increases. The high cooling rate results in a higher temperature gradient at the leading edge of the solid–liquid interface, thereby leading to more heterogeneous nucleation and inhibiting the grain growth^16^. Numerous solidification studies have been developed to correlate the cooling rate with SDAS [16, 20, and 23]. The average cooling rate is correlated with SDAS as below: SDAS = K (CR)-n where K and n are constants for a fixed alloy composition and CR is the average cooling rate. For Al-13Si-4Cu-1 Mg-2Ni alloy studied in Ref.^16^ using power-law regression analysis, a quantitative relationship between the cooling rate (*v*) and the SDAS (D) of the alloy can be obtained by fitting the data: D = 47 (*v*)-1/3. This relation is very close to the relationship developed by Ref.^24^ that SDAS in A356/A357 alloys fits well with the empirical equation SDAS = 39.4R-0.317, where R represents the mean cooling rate of primary α(Al) dendrites during solidification. In the present investigation the equation of ref. 24 was taken to calculate the average cooling rate for Al-17wt% and Al-20wt% alloys. Table 3 gives the calculated cooling rate of each processed sample according to the formula in Ref.^23^. In Ref. 16, the average cooling rate for SDAS 0.83 µm was 1.5 × 10^5^ °C/s.

**Table 3:** Average cooling rates versus SDAS of Al-17wt.% and Al-20wt.%Si alloys.

Sample no.	Alloy	Melted depth, mm	Average SDAS, µm	Average calculated cooling rate, ^o^C/s ^24^
A#1	Al− 17%Si	3	2.5	6 × 10^3^
A#2	Al− 17%Si	0.5	1.5	3 × 10^4^
A#3	Al− 17%Si	0.3	1	1 × 10^5^
A#4	Al− 17%Si	0.16	0.5	1 × 10^6^
B#1	Al− 20%Si	3	1	1 × 10^5^
B#2	Al− 20%Si	0.45	0.5	1 × 10^6^
B#3	Al− 20%Si	0.25	0.25	3 × 10^6^

#### The inter-lamellar spacing λ

**Figure 13:**
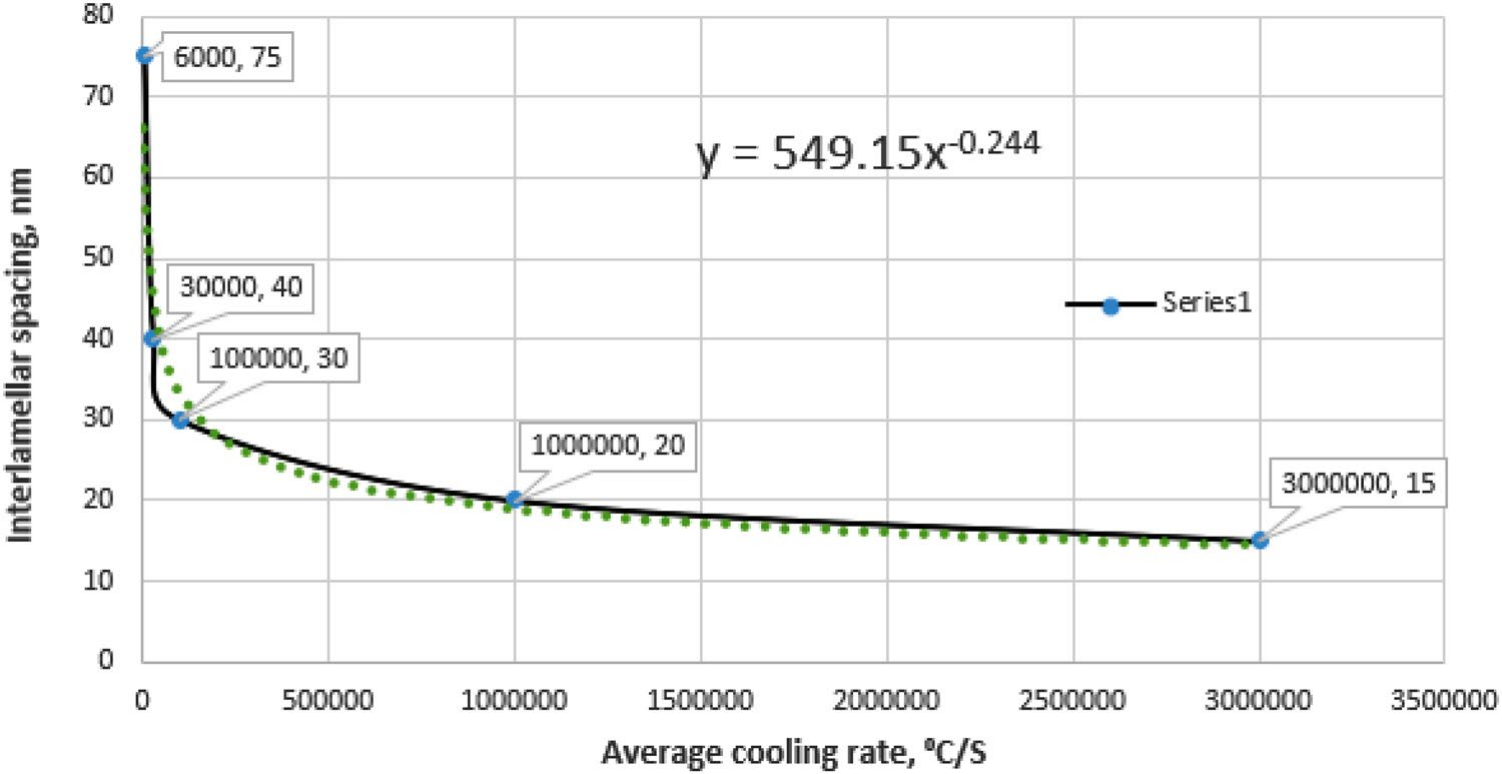
The relationship between average cooling rate and interlamellar spacing for Al-17wt.% Si alloy.

In the present work, the inter-lamellar spacing λ of the eutectic Si of various specimens that are processed at different conditions were measured from the SEM and correlated with the cooling rate and summarized in Table 4. From this data, the relationship between the average cooling rate and interlamellar spacing λ for the Al-17wt.%Si and Al-20wt.%Si alloys can be drawn (Fig. 13). It is clear from the figure that the interlamellar spacing is inversely proportional to the cooling rate and also is dependent on the composition. It seems that the relationship between cooling rate and interlamellar spacing obeys this formula λ = 550 (CR)-^0.25^ for Al-17wt.%Si alloy.

**Table 4:** Silicon morphology and eutectic spacing as a function of processing variables.

Sample no.	Laser power W	Scanning speed, mm /s	Melted depth, mm	Si morphology	Average cooling rate, ^o^C/s ^24^	λ, nm
A#1	2,500	5	3	Flakes, feathery and fibrous	6 × 10^3^	50–100
A#2	1,800	10	0.5	Broad, flaky	3 × 10^4^	30–50
A#3	1,800	25	0.3	Chain like and fibrous	1 × 10^5^	30
A#4	2,500	100	0.16	Elongated flake, Fibrous, and agglomerated rounded particles	1 × 10^6^	20
B#1	2,500	5	3	Mixed fibrous and flakes	1 × 10^5^	30
B#2	2,500	50	0.45	Y, chain like and thread like fibrous	1 × 10^6^	20
B#3	2,500	100	0.25	Chain like and thread like fibrous	3 × 10^6^	10

Under different cooling conditions, it can be observed that with increasing cooling rate, the eutectic silicon size refines, and the branched morphology becomes more apparent. When the cooling rate increased to more than 105 oC/s, the flaky or lamella shape eutectic silicon disappeared and the branched structure of fibrous shape enhanced, weakening eutectic silicon facet characterization. In general, the shift of the growth direction causes anisotropic growth which shortens a Si crystal in the initial growth direction and increases its thickness in the lateral direction (Figs. 3, 4, 5, 6).

#### X-ray diffraction analysis

**Figure 14:**
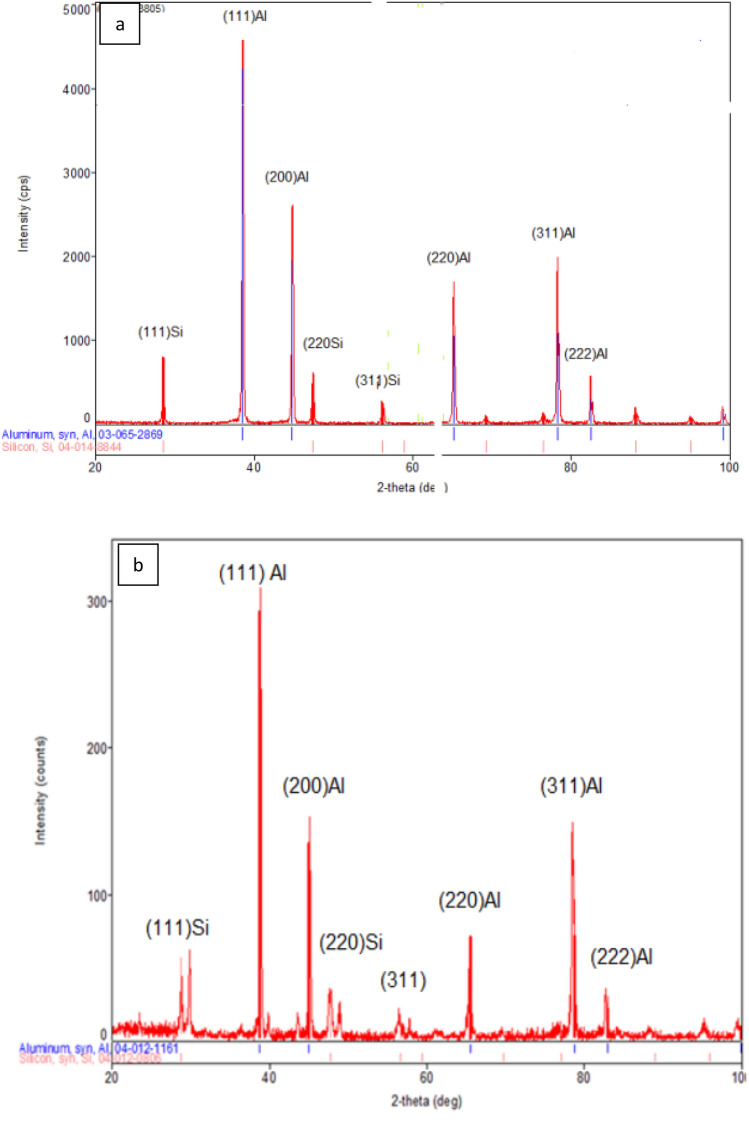
XRD taken from **a** the surface of as-received Al-20wt.%Si, **b** the top surface of laser melted zone of Al-20wt.%Si alloy (2,500 W, 1 mm, and 50 mm/s, liquid nitrogen immersed).

XRD patterns taken from the surface of the as-received Al-20%Si alloy and from the top surface of the LMZ of the same alloy (processing condition: 2,500 W, 50 mm/s, liquid nitrogen) are shown in Fig. 14a,b. Calculated d spacing values for aluminum and silicon before and after laser melting are given in Table 5. The analysis of the position of peaks, d-spacing, and lattice parameters of the α-aluminum showed a clear shift in the position of α-Al towards the higher diffracting angle and consequently a decrease in d spacing and lattice parameter (Fig. 14a,b). For example, the d spacing of the maximum intensity peak (111) of aluminum was 2.3341 Å. After laser melting it is reduced to 2.319 Å (∆d = 0.0151). Calculated d spacing values for the as-received and LMZ samples are included in Table 5. The lattice constant of aluminum (a) was calculated and is reduced from 4.0427 nm to 4.0167 (∆a = 0.026). This decrease in the d spacing and lattice parameter of aluminum is probably due to the increased solubility of silicon in aluminum-induced by the rapid cooling during solidification.

**Table 5:** 2-theta and corresponding d spacing of as received and laser melted of Al-20%Si alloy.

As – received alloy		*hkl*	Laser melted alloy	
2-theta (degree)	d-spacing		2-theta (degree)	d-spacing
28.542	3.1248	(111)Si	28.730	3.1048
38.540	2.3341	(111)Al	38.799	2.3191
44.810	2.0210	(200)Al	45.106	2.0084
47.413	1.919	(220)Si	47.708	1.9048
56.231	1.6346	(311)Si	48.862	1.8624
65.141	1.43088	(220)Al	65.396	1.4259
78.249	1.2207	(331)Al	78.788	1.2176
82.461	1.16873	(222)Al	82.72	1.1658
88.119	1.1077	(422)Si	No peak	No peak
95.00	1.0448	(511)Si	95.27	1.0425
99.111	1.01217	(400)Al	99.41	1.0100

#### Microhardness measurements

**Figure 15:**
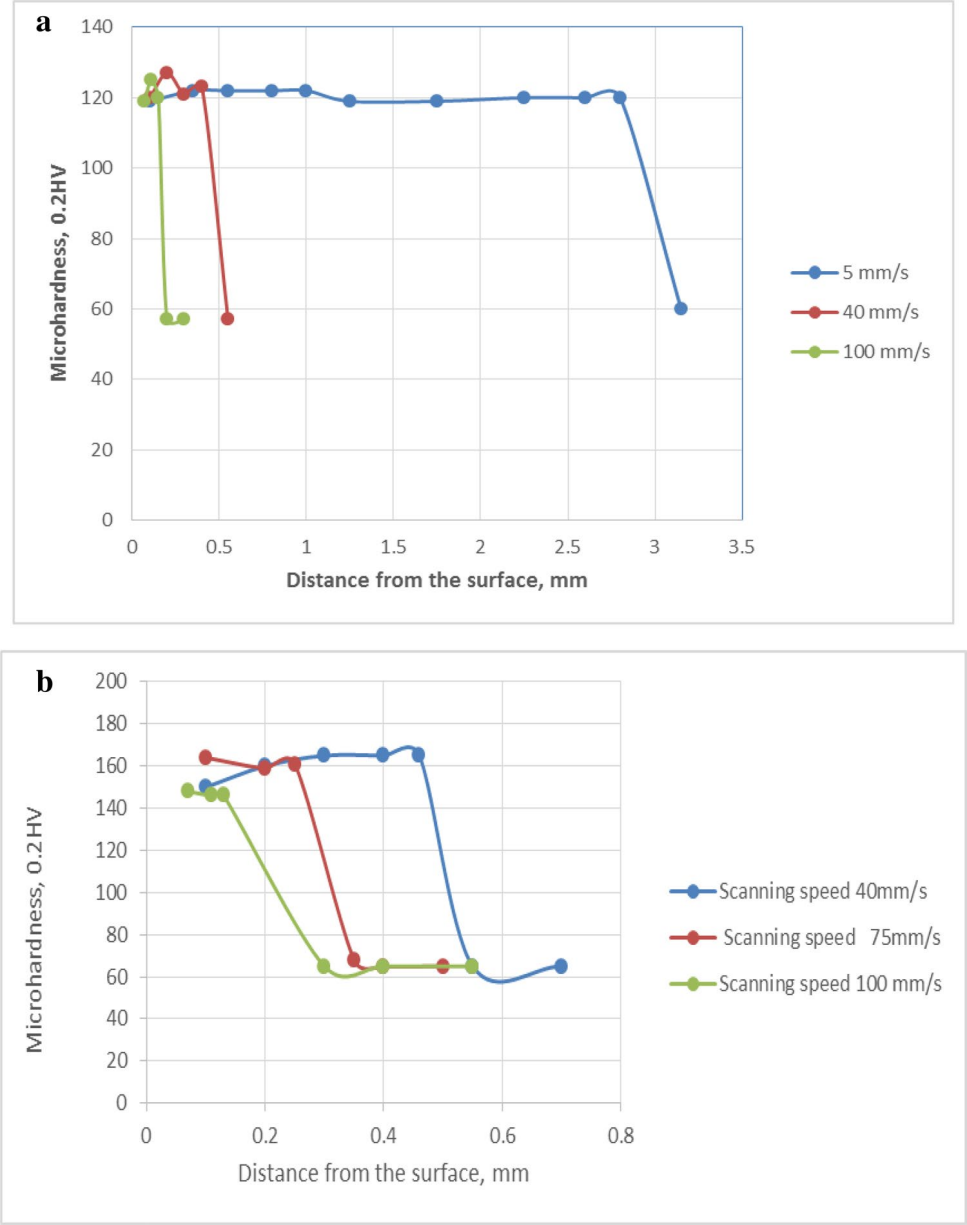
**a** Microhardness profile across the depth for three laser melted samples of Al-17wt.%Si alloys processed at different scanning speeds. **b** Microhardness profile across the depth of three laser melted samples of Al-20wt.%Si alloys immersed in liquid nitrogen and laser melted at different scanning speeds.

The microhardness profiles across the LMZ of Al-17wt.%Si and Al–20wt.%Si alloys under various processing conditions are shown in Fig. 15. A two-fold increase in microhardness of the alloy is observed after laser melting from 60 to 120 HV (± 5). This hardness value is the average value of α Al dendrites and the eutectic. It seems from these curves that the hardness is independent of the scanning speed. Furthermore, a higher microhardness value of 160 HV (± 5) was shown in the Al-20wt.%Si alloy supercooled in liquid nitrogen followed by laser melting. This represents a three-fold increase in hardness with increasing cooling rate. To measure the microhardness in a specific area i.e. in the eutectic zone, it is necessary to use a micro-indenter that is capable of measuring the hardness in the eutectic area. Table 4 gives the indentation hardness of Al-20wt.%Si at different locations in the base metal and the melted zone. Increasing cooling rate reduced the interlamellar eutectic spacing and hence increased the hardness.

#### Indentation hardness measurements

Indentations were performed on different locations in the LMZ and the substrate to measure hardness variation of the eutectic zone of Al-20wt.%Si alloy (0.5 mm deep) and to measure hardness variation within different eutectic regions. A Berkovich indenter on a high load transducer was employed with a peak load of 850 μN and a quasi-static trapezoidal loading function (5 s load and unload 2 s hold time).

**Figure 16:**
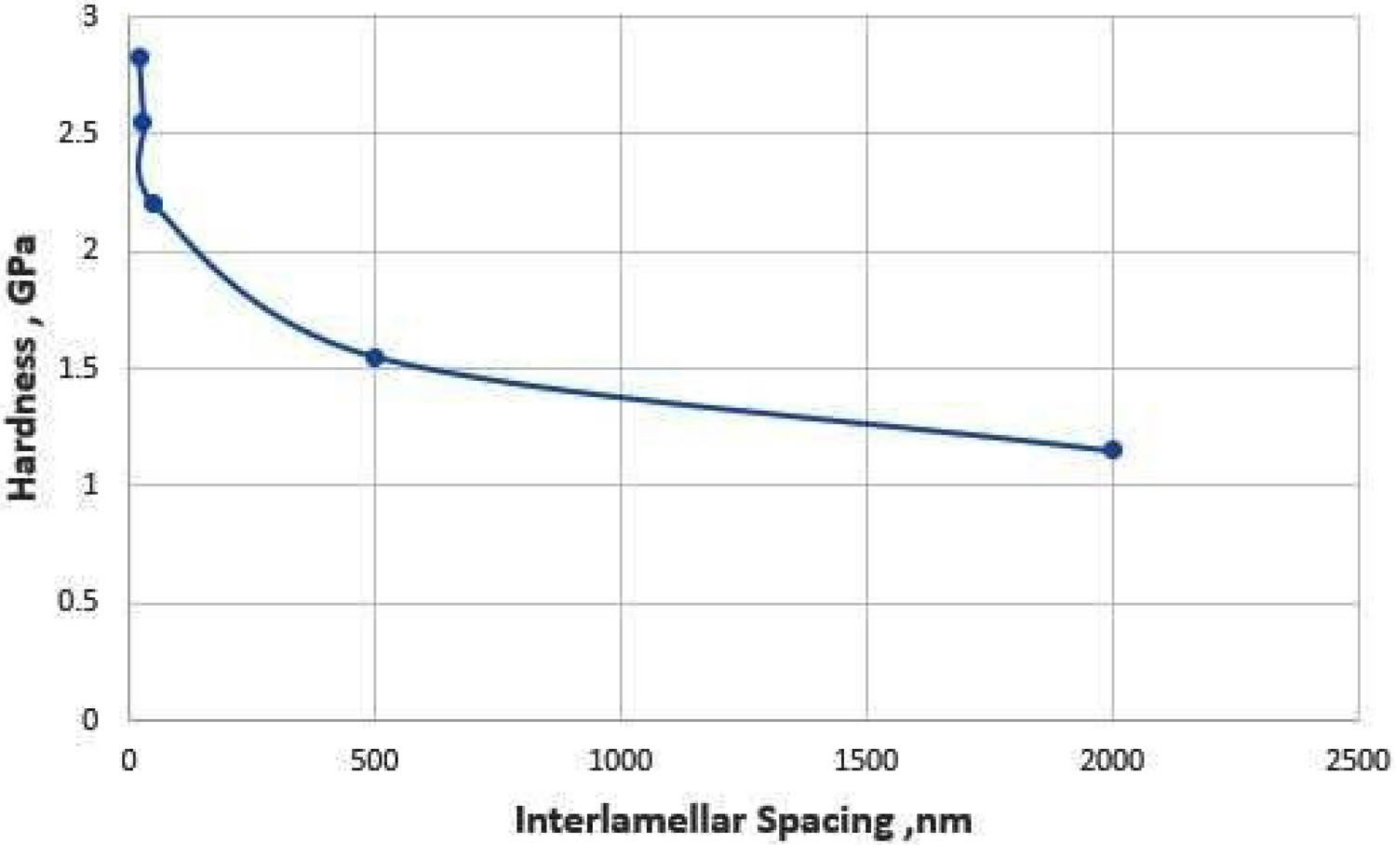
Hardness versus interlamellar spacing of Al-17wt.%Si alloy.

Figure 16 shows the variations of hardness (H) and average interlamellar spacing (λ). The hardness increased with a decreasing interlamellar spacing λ. The hardness values were 2.83 GPa, 2.55 GPa, 2.2 GPa, 1.55GPa, and 1.15GPa and the corresponding values of the interlamellar spacing were 20 nm, 30 nm, 50 nm, 2000 nm, and 5,000 nm respectively.

## Discussion

Solidification of hypereutectic alloys under slow cooling rate begins at the liquidus temperature with precipitation, of course, plate-like Si-particles as the primary phase. Further cooling to the temperature of the eutectic transformation leads to a cooperative growth mechanism between Si and Al, and finally to the formation of the eutectic in the remaining part of the melt pool. In contrast, a higher cooling rate shifts the eutectic composition towards higher silicon content and depresses the eutectic temperature. As a result, the nominal hypereutectic alloy can have the solidification structure of a hypoeutectic or a eutectic alloy. The mechanism of refinement can be described based on the surface energy of the Al–Si solid interface^29^. This theory states that the rate of advance of the solidification interface depends on a balance between the rate of heat flow from the liquid to the solid through the interface and the latent heat of fusion released during solidification. The thermal conductivities of Al and Si in their pure form are 205 and 83 W/ (mK) respectively, and their latent heats of fusion are 396 and 1,411 J/g respectively. Since the difference between the magnitude of the thermal conductivity of pure Al and pure Si and the difference between the magnitude of the latent heat of fusion of pure Al and pure Si are large, Al will solidify much faster than Si. Thus, Al gains a lead during the solidification of the eutectic. As the cooling rate increases, the lead of Al over Si increases causing complete encasement of the lagging Si crystal by the advancing Al. This theory accounts for the formation of the modified eutectic structure at high cooling rates. In some investigations about the quenched structure of directionally solidified Al–Si alloys reveal that silicon leads at the interface^33^ while growing in a very faceted manner. However, in some regions, silicon and aluminum were found to be growing together.

Regarding the role of twins in the modification of silicon in the Al–Si alloys, extensive studies have been carried out by different research groups, e.g. Hogan^27^, Lu, and Hellawell^28,29^, Dahle ^30^ Nogita et al.^31^. Lien et al.^34^, and Lu and Hellawell^28,29^ reported that twin density in the unmodified silicon flakes is very low while the modified silicon by trace additions showed a massive increase in the twins. Silicon is a semi-metal and solidifies in a faceted manner. For the growth of the faceted face, there will be kinetic limitations to a molecular attachment on certain crystal faces notably closely packed planes and that the presence of internal defects such as twins can provide important sites for molecular attachment giving rise to re-entrant edges and grooves on growing surfaces which are self-perpetuating^31^. Nogita et al. explain the mechanism of the twinning of silicon in modified alloys by suggesting that atoms of the modifier are absorbed onto the growth steps of the silicon solid–liquid interface. consistent with the impurity induced twinning model^31^.

Based on TEM results (Figs.10, 11, 12), a high density of twins can be considered as a basis for explaining the modification effects in the laser melted zone of the Al-17wt.%Si and Al-20wt.%Si alloys. The highly twinned silicon particles which resulted from the high cooling rates offer ways to create more step or defects to lower the total surface energy of the system, Also, it provides new growth direction and consequently leads to bending and branching. It has been proposed by several authors^26–31^ that branching of silicon particles is due to significant growth differences between [111] and [112] directions since growth in [111] direction is more difficult than in [112] direction. High cooling rate resulted in an increase in the growth speed in [112] direction and consequently causes the formation of curved and refined silicon crystals.

The presence of the rounded and clustered silicon particles near and around the silicon eutectic is probably a product of a divorced eutectic which followed the eutectic solidification. The aluminum dendrite and the aluminum eutectic are highly saturated with the silicon and as the temperature decreases, the solubility decreases, and more silicon crystals will form.

## Conclusions

The following conclusions were drawn from this work:

1. Laser surface remelting of Al-17wt%Si and Al-20wt% Si alloys has led to the formation of hypoeutectic structures comprising of α (Al) dendrites surrounded by a lamellar eutectic of irregular morphology.
2. The secondary dendritic arm spacing SDAS of the (α-Al) decreased from 3 µm to 0.5 µm and the interlamellar eutectic spacing (λ) reduced from 10,000 nm to 30 nm as the cooling rate increased from 6 × 10^3^ °C/s to 3.3 × 10^6^ °C/s..
3. There was a significant change in silicon morphology from massive and plate/needle-like to a mixture of round-cornered flakes and fibrous. All the re-solidified silicon particles were severely branched and had a coral-like or thread-like morphology.
4. Most of the silicon crystals in the laser melted zone were heavily twinned and partially faceted.
5. Conventional microhardness measurements and indentation probe microhardness showed a 2–threefold increase in microhardness. The finest interlamellar spacing (λ) showed the highest indentation hardness value.
6. Rapid cooling led to a significant increase in the solid solubility of silicon in aluminum to about 5wt.% compared to 0.5wt.% in slow cooling.
